# Intervening in hnRNPA2B1-mediated exosomal transfer of tumor-suppressive miR-184-3p for tumor microenvironment regulation and cancer therapy

**DOI:** 10.1186/s12951-023-02190-w

**Published:** 2023-11-14

**Authors:** Xueqing Zhou, Yiling Hong, Yupeng Liu, Li Wang, Xuan Liu, Yi Li, Hong Yuan, Fuqiang Hu

**Affiliations:** 1 College of pharmaceutical science, Zhejiang University, Hangzhou, 310058 China; 2 Department of Clinical Pharmacology, Affiliated Hangzhou First People’s Hospital, School of Medicine, Zhejiang University, Hangzhou, 310006 China; 3 Jinhua Institute of Zhejiang University, Jinhua, 321299 China

**Keywords:** TNBCs, TAMs, Exosomes, miR-184-3p, hnRNPA2B1, Nanoparticle

## Abstract

**Background:**

Despite being a common malignant tumor, the molecular mechanism underlying the initiation and progression of triple-negative breast cancers (TNBCs) remain unclear. Tumor-associated macrophages (TAMs) are often polarized into a pro-tumor phenotype and are associated with a poor prognosis of TNBCs. Exosomes, important mediators of cell-cell communication, can be actively secreted by donor cells to reprogram recipient cells. The functions and molecular mechanisms of tumor cell-derived exosomes in TNBCs progression and TAMs reprogramming urgently need to be further explored.

**Results:**

We demonstrated that tumor cell-derived exosomes enriched with miR-184-3p were taken up by macrophages to inhibit JNK signaling pathway by targeting EGR1, thereby inducing M2 polarization of macrophages and synergistically promoting tumor progression. Nanoparticles loaded with oncogene *c-Myc* inhibitor JQ1 could suppress the polarization process by reducing Rac1-related exosome uptake by macrophage. More importantly, it was found for the first time that tumor-suppressive miR-184-3p was actively sorted into exosomes by binding to RNA-binding protein heterogeneous nuclear ribonucleoprotein A2B1 (hnRNPA2B1), thus facilitating tumor cell proliferation and metastasis by relieving the inhibitory effect of miR-184-3p on Mastermind-like 1 (MAML1). Overexpressing miR-184-3p in tumor cells and simultaneously knocking down hnRNPA2B1 to block its secretion through exosomes could effectively inhibit tumor growth and metastasis.

**Conclusions:**

Our study revealed that hnRNPA2B1-mediated exosomal transfer of tumor-suppressive miR-184-3p from breast cancer cells to macrophages was an important mediator of TNBCs progression, providing new insights into TNBCs pathogenesis and therapeutic strategies.

**Graphical Abstract:**

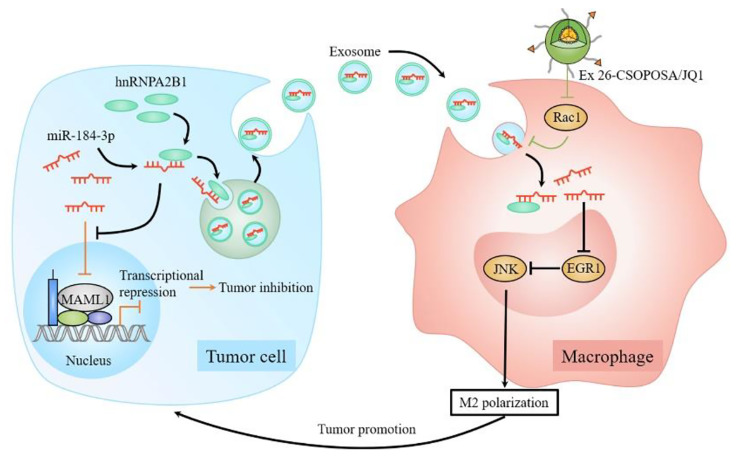

**Supplementary Information:**

The online version contains supplementary material available at 10.1186/s12951-023-02190-w.

## Background

At present, female breast cancer is the most commonly diagnosed cancer and the leading cause of cancer death [1]. Triple-negative breast cancers (TNBCs), the most aggressive subtype of this disease, are associated with increased risk of developing metastatic disease and reduced survival rates [2, 3]. Despite being a common malignant tumor, the molecular mechanism underlying the initiation and progression of TNBCs still urgently need to be further explored to identify new targets for the development of novel therapeutic strategies against TNBCs [4].

A key reason for the malignancy of TNBCs is its unique tumor microenvironment (TME), which is characterized by the infiltration of a large number of immunosuppressive cells, such as tumor-associated macrophages (TAMs) [5]. Multiple independent investigations have found that the increased abundance of TAMs is generally associated with a poorer prognosis [6, 7]. As the most abundant inflammatory cells, macrophages can be polarized into classically activated (M1) or alternatively activated (M2) subtypes responding to environmental signals [8]. They differ in the expression and function of receptors, cytokines, and chemokines. M2 macrophages, marked by CD206, typically express high levels of anti-inflammatory cytokines (e.g., IL-10), growth factors and protease to support their pro-tumor functions [9, 10]. TAMs are more likely to polarize into M2 macrophages, which play a role in many aspects of tumor progression [11]. The mechanism of macrophage polarization in TNBCs still needs to be further explored.

Exosomes, nano-sized bilayer vesicles secreted by most cells, are important mediators of cell-cell communication [12, 13]. Exosomes are rich in proteins, messenger RNAs (mRNAs), microRNAs (miRNAs), and other important biomolecules [14]. When exosomes are taken up by other cells, these biomolecules can be transferred to recipient cells and reprogram recipient cells by regulating signaling pathways [15]. MiRNAs, a family of endogenous non-coding tiny RNA molecules, have recently attracted great attention in cancer research. They play a central role in gene expression regulation by targeting mRNA degradation and inhibiting protein translation. Studies have shown that miRNAs can function as oncogenes or tumor suppressors, and the dysregulation of miRNAs is related to the occurrence and development of many cancers [16]. More importantly, miRNAs play a role not only inside cells but also in the TME by regulating the expression of specific genes [17, 18]. As some studies have demonstrated, miRNAs in exosomes can function as secreted signaling molecules affecting recipient cells [19, 20]. Exosomes derived from different cells contain unique miRNA expression profiles, which may differ from the signatures of their parent cells [21, 22]. Therefore, we tried to reveal the functions and molecular mechanisms of the specific miRNA in TNBCs.

In this study, the role of tumor cell-derived exosomes in macrophage polarization was studied in vitro and in vivo. RNA sequencing and bioinformatics analysis were used to screen the key exosomal miRNA and explore the molecular mechanisms of its effects on macrophage polarization and TNBCs progression. Meanwhile, we used the previously designed ROS-sensitive micelles modified with the sphingosine 1-phosphate receptor 1 (S1PR1) antagonist Ex 26 (Ex 26-CSOPOSA) to deliver the oncogene *c-Myc* inhibitor JQ1 (Ex 26-CSOPOSA/JQ1) *via* S1PR1 on macrophages [23], and investigated the intervention effect of Ex 26-CSOPOSA/JQ1 on exosome uptake by macrophages. The molecular mechanism by which the involved miRNA was sorted into exosomes was further investigated, providing a new approach for the treatment of TNBCs.

## Results

### Tumor cell-derived exosomes induced M2 polarization of macrophages, which subsequently promoted the proliferation and migration of tumor cells

Exosomes are critical mediators of intercellular communication in the TME. 4T1 cell-derived exosomes (T-exo) was extracted, and the characterization of exosomes was confirmed. The particle analyzer revealed that the particle size of the extracted exosomes was 75.9 ± 3.56 nm with the typical cup-shaped morphology showed by transmission electron microscopy (TEM) (Additional file 1: Fig. S1a, b). The results of western blot (Additional file 1: Fig. S1c) showed that the exosomes highly expressed CD63, a known exosomal marker, while the expression of CD63 in the corresponding cell lysate was low.

**Fig. 1:**
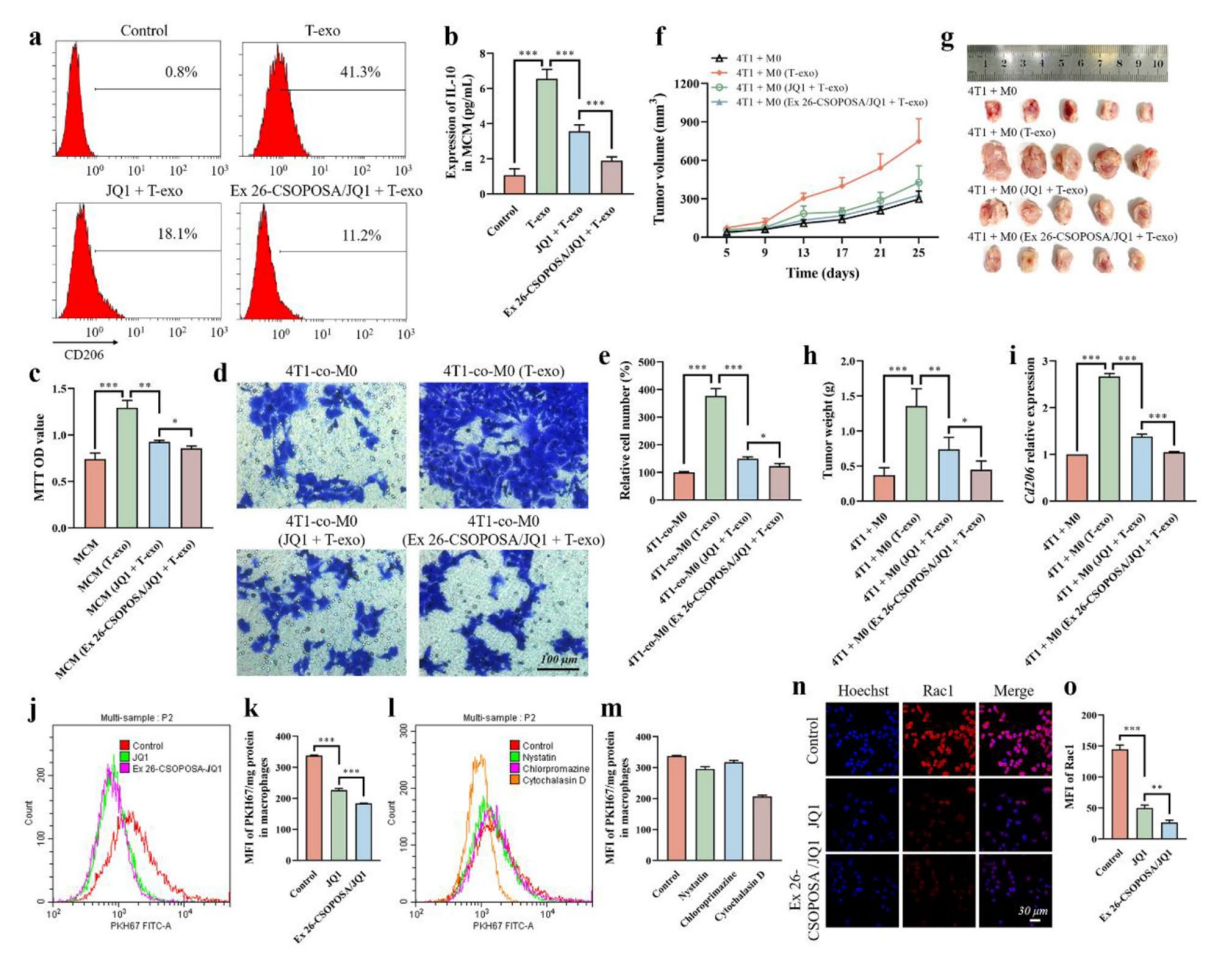
T-exo promoted tumor progression by inducing M2 macrophage polarization, which was blocked by Ex 26-CSOPOSA/JQ1. FCM for CD206 levels **(a)** and ELISA for the secretion of IL-10 **(b)** of T-exo-stimulated macrophages with or without the pretreatment of JQ1 or Ex 26-CSOPOSA/JQ1 (n = 3). **c** The proliferation of 4T1 cells cultured with MCM from macrophages treated as described above assessed by MTT assay (n = 3). Representative images of transwell migration assay **(d)** and the relative migration cell number **(e)** of 4T1 cells co-cultured with M0 macrophages (4T1-co-M0) treated as described above (n = 3). Tumor volume changes **(f)**, image of tumors **(g)** and tumor weight **(h)** of each group (n = 5). **i** The expression of *Cd206* in tumor tissues detected by qRT-PCR (n = 3). The fluorescence intensity of PKH67 measured by FCM **(j)** and fluorescence spectrophotometry **(k)** in macrophages after incubated with PKH67-labelled T-exo with or without the pretreatment of JQ1 or Ex 26-CSOPOSA/JQ1 (n = 3). The uptake mechanism of PKH67-labelled T-exo by macrophages tested by FCM **(l)** and fluorescence spectrophotometry **(m)** by pretreating cells with inhibitors of different internalization pathways. **n** The levels of Rac1 in macrophages determined by IF staining. Cell nuclei were blue. Rac1 was red. **o** MFI of Rac1 in (**n**) calculated by ImageJ software (n = 3). Data were expressed as mean ± SD (*p < 0.05, **p < 0.01, ***p < 0.001)

To determine whether T-exo could induce M2 polarization of macrophages, macrophages were treated with T-exo and the expression of recognized macrophage marker CD206 (Fig. 1a) and secreted IL-10 in macrophage-conditioned medium (MCM) (Fig. 1b) was detected by flow cytometry (FCM) and enzyme-linked immunosorbent assay (ELISA), respectively. The results showed that the expression levels of CD206 and IL-10 were highly increased in macrophages treated with T-exo. Meanwhile, we wondered whether the oncogene *c-Myc* might have an effect on this process. Due to the insolubility and systemic toxicity of *c-Myc* inhibitor JQ1, we previously designed and prepared JQ1-loaded nanoparticles (Ex 26-CSOPOSA/JQ1), which possessed S1PR1-targeting and ROS-sensitive release characteristics, to improve the function of JQ1. After pretreatment with JQ1 or Ex 26-CSOPOSA/JQ1, we found that the expression of CD206 and IL-10 induced by T-exo stimulation was significantly down-regulated. And compared with free JQ1, Ex 26-CSOPOSA/JQ1 exhibited a stronger inhibitory effect. Observing the morphological changes of macrophages in this process (Additional file 1: Fig. S2), it could be seen that the macrophages co-incubated with T-exo showed a long spindle type, which was a typical M2 polarization form. JQ1 and Ex 26-CSOPOSA/JQ1 pretreatment could maintain the sphericity of macrophages.

Furthermore, we investigated the role of T-exo-polarized macrophages in tumor cell proliferation and migration. After 4T1 cells were cultured with different MCM for 24 h, the results of MTT assay (Fig. 1c) showed that the conditioned medium from T-exo-treated macrophages (MCM (T-exo)) significantly promoted the proliferation of 4T1 cells, while the pretreatment of JQ1 and Ex 26-CSOPOSA/JQ1 could effectively inhibit this promoting effect. The transwell assay evaluated the migration of 4T1 cells induced by different conditioned macrophages and showed the same trend (Fig. 1d, e).

To further investigate the tumor-promoting effect of polarized macrophages in vivo, 4T1 cells mixed with M0 macrophages (4T1 + M0) or T-exo-polarized macrophages pre-treated with or without JQ1 or Ex 26-CSOPOSA/JQ1 were in situ inoculated into Balb/c mice. The tumor growth curve and tumor weight on the last day (Fig. 1f-h) were measured, and the expression of *Cd206* in tumor tissues (Fig. 1i) was measured by quantitative real-time PCR (qRT-PCR). Consistent with the previous results, 4T1 cells mixed with T-exo-polarized macrophages generated larger and heavier tumors with high expression of *Cd206*. Pretreating macrophages with JQ1 or Ex 26-CSOPOSA/JQ1 could slow the tumor growth and down-regulate the expression of *Cd206*. Collectively, these results demonstrated that T-exo could induce M2 polarization of macrophages to accelerate tumor progression, and Ex 26-CSOPOSA/JQ1 could inhibit the tumor-promoting effect of T-exo by interfering with the polarization process.

### Ex 26-CSOPOSA/JQ1 reduced the uptake of tumor cell-derived exosomes by macrophages by down-regulating the expression of Rac1

To explore the mechanism by which Ex 26-CSOPOSA/JQ1 interfered with T-exo-induced macrophage polarization, we focused on the effect of Ex 26-CSOPOSA/JQ1 on exosome uptake by macrophages. Macrophages were incubated with PKH67-labelled exosomes for 2 h, and the fluorescence intensity of PKH67 was measured by FCM (Fig. 1j) and fluorescence spectrophotometry (Fig. 1k). Compared with the control group, after incubating JQ1 or Ex 26-CSOPOSA/JQ1 with cells for 24 h, the mean fluorescence intensity (MFI) of PKH67 was significantly reduced, indicating that the uptake of exosomes by macrophages was significantly inhibited.

Therefore, we further investigated the mechanism of exosome uptake. Nystatin, Chlorpromazine and Cytochalasin D were used as specific inhibitors of Caveolin-mediated endocytosis, Clathrin-mediated endocytosis and micropinocytosis, respectively. The results detected by FCM (Fig. 1l) and fluorescence spectrophotometry (Fig. 1m) showed that the uptake of T-exo by macrophages was mainly inhibited by Cytochalasin D, indicating that it was mainly through macropinocytosis pathway, Therefore, we hypothesized that Rac1, a key protein in the macropinocytosis pathway [24, 25], might be a key molecule by which JQ1 and Ex 26-CSOPOSA/JQ1 inhibited the exosome uptake. Then, immunofluorescence (IF) staining was performed to determine the expression of Rac1 on macrophages, and the results showed that JQ1 and Ex 26-CSOPOSA/JQ1 significantly down-regulated the expression level of Rac1 (Fig. 1n, o). All these results suggested that Ex 26-CSOPOSA/JQ1 reduced the uptake of T-exo by macrophages by down-regulating the expression of Rac1, thereby interfering with T-exo-induced M2 polarization of macrophages.

### MiR-184-3p enriched in tumor cell-derived exosomes was delivered to macrophages to induce M2 polarization of macrophages and promote tumor progression

**Fig. 2:**
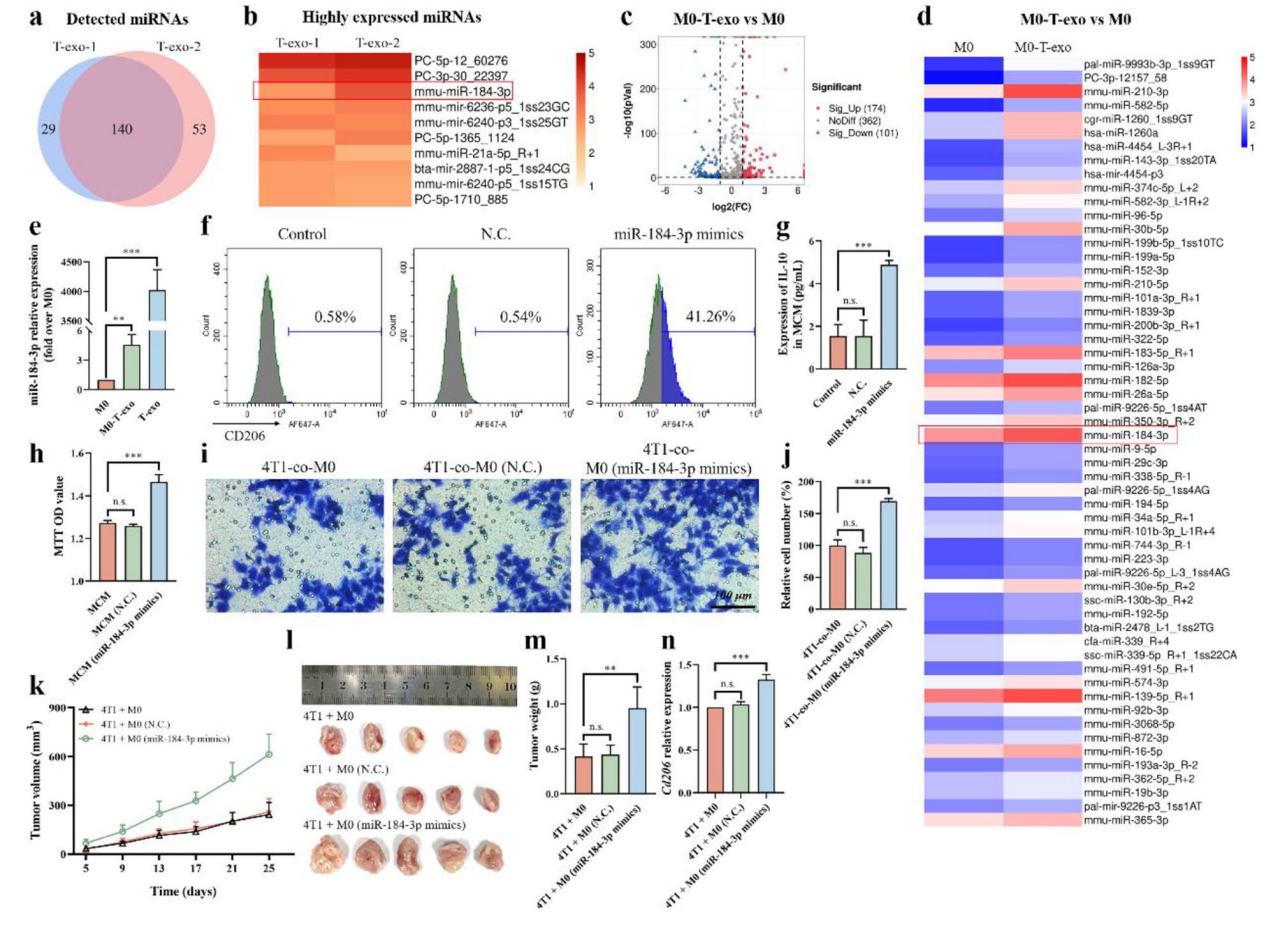
MiR-184-3p enriched in T-exo was delivered to macrophages to promote tumor progression. **a** Venn diagram showed the overlap of miRNAs in two batches of T-exo detected by miRNA-seq. **b** Heatmap of the top 10 highly expressed miRNAs in (**a**). **c** Volcano of differentially expressed miRNAs in macrophages treated with or without T-exo detected by miRNA-seq. **d** Heatmap of the up-regulated miRNAs with high and middle expression in (**c**). **e** The expression of miR-184-3p in macrophages and T-exo detected by qRT-PCR (n = 3). CD206 levels **(f)** and secreted IL-10 **(g)** of macrophages transfected with negative control (N.C.) or miR-184-3p mimics (n = 3). **h** The proliferation of 4T1 cells cultured with MCM from macrophages treated as described above assessed by MTT assay (n = 3). Representative images of transwell migration assay **(i)** and the relative migration cell number **(j)** of 4T1 cells co-cultured with macrophages treated as described above (n = 3). Tumor volume changes **(k)**, image of tumors **(l)** and tumor weight **(m)** of each group (n = 5). **n** The expression of *Cd206* in tumor tissues detected by qRT-PCR (n = 3). Data were expressed as mean ± SD (**p < 0.01, ***p < 0.001)

MiRNAs have been reported to be important cargos, which can be sorted into exosomes and delivered to target cells, thereby reprogramming the phenotype of target cells. In order to clarify whether miRNAs play a key role in the M2 polarization process, miRNA sequencing (miRNA-seq) of two batches of T-exo and macrophages treated with or without T-exo was performed to analyze the miRNA profiles in T-exo and the differentially expressed miRNAs in macrophages under different treatments. Among the miRNAs identified, 140 overlapping miRNAs were identified and confirmed in two batches of T-exo (Fig. 2a), and we focused on the top 10 highly expressed miRNAs (Fig. 2b). Compared to M0 macrophages, 174 miRNAs were up-regulated and 101 miRNAs were down-regulated in T-exo-treated M0 macrophages (M0-T-exo) (Fig. 2c). We further screened the up-regulated miRNAs with high and middle expression and found that miR-184-3p highly expressed in T-exo was significantly up-regulated in M0-T-exo (Fig. 2d).

In line with this finding, high expression of miR-184-3p was detected in T-exo and M0-T-exo by qRT-PCR compared with M0 macrophages (Fig. 2e), indicating that miR-184-3p could be delivered to macrophages *via* T-exo. In addition, pretreatment with JQ1 or Ex 26-CSOPOSA/JQ1 could reduce the expression of miR-184-3p up-regulated by T-exo in macrophages (Additional file 1: Fig. S3). Therefore, we sought to determine whether miR-184-3p was the key molecule in T-exo-induced M2 polarization of macrophages. After transfection with miR-184-3p mimics to up-regulate the expression of miR-184-3p in macrophages (Additional file 1: Fig. S4), it was found that M2 macrophage markers, including CD206 and IL-10, were significantly induced to increase (Fig. 2f, g). Furthermore, MCM and corresponding transfected macrophages were collected for MTT assay (Fig. 2h) and transwell assay (Fig. 2i, j) described above to evaluate the effect on proliferation and migration of tumor cells. Apparently, both the proliferation and migration abilities of 4T1 cells were significantly increased by macrophages transfected with miR-184-3p mimics.

We further validated the function of miR-184-3p in vivo by inoculating 4T1 cells mixed with macrophages transfected with miR-184-3p mimics or negative control (N.C.) into Balb/c mice. The tumor volume and tumor weight were measured (Fig. 2k-m), and the expression of *Cd206* in tumor tissues was measured by qRT-PCR (Fig. 2n). The results showed that mice in 4T1 + M0 (miR-184-3p mimics) group bore with larger and heavier tumors infiltrated with more M2 macrophages. Altogether, these results demonstrated that miR-184-3p was enriched in T-exo, which could be delivered to macrophages *via* exosomes to induce M2 polarization of macrophages, thereby promoting tumor cells proliferation and migration.

### Exosomal miR-184-3p induced M2 polarization of macrophages ***via*** down-regulating EGR1 expression and inhibiting the JNK signaling pathway

**Fig. 3:**
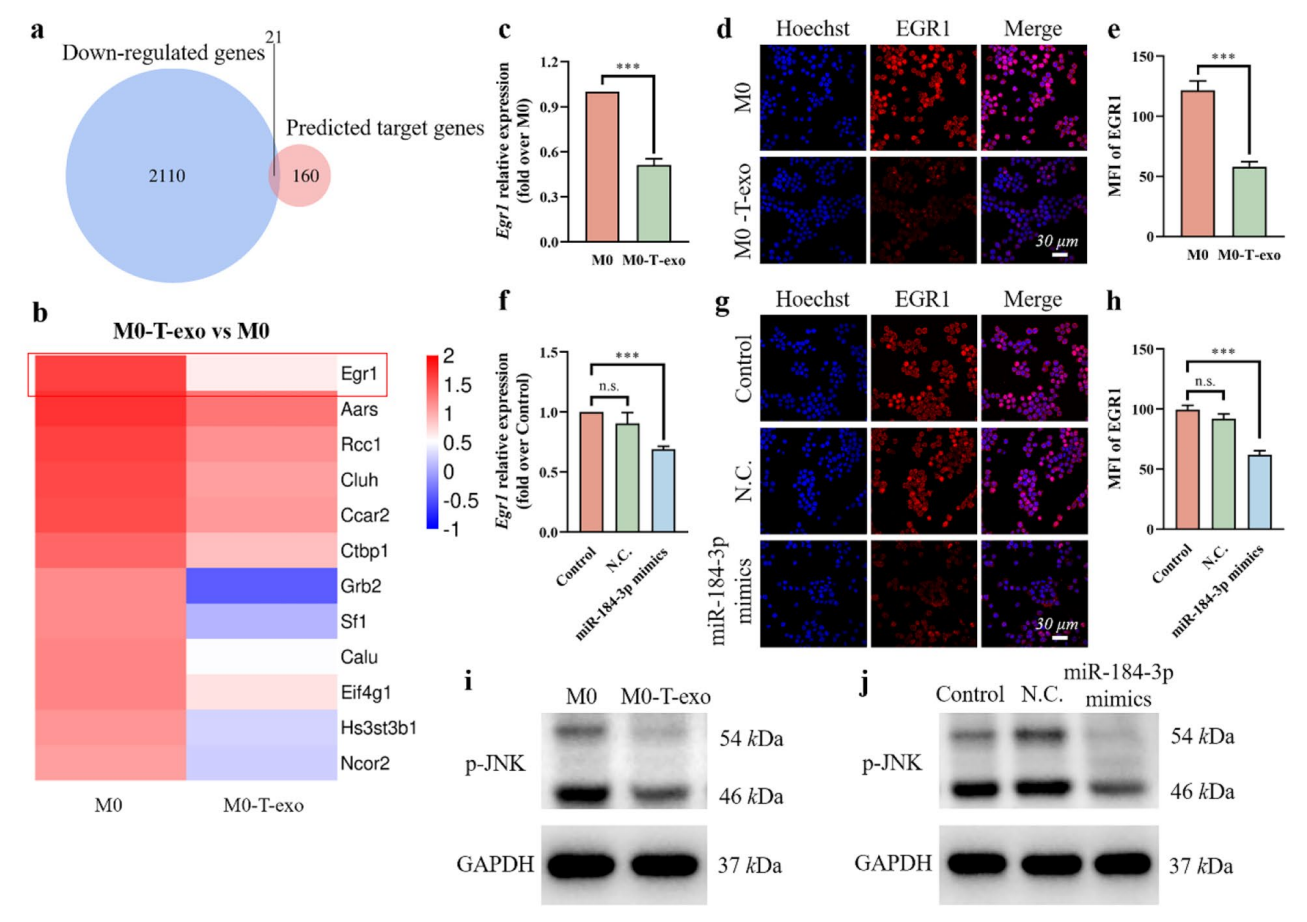
Exosomal miR-184-3p induced M2 macrophage polarization by inhibiting EGR1 expression and JNK signaling pathway. **a** Venn diagram showed the overlap of mRNAs in down-regulated genes in M0-T-exo detected by mRNA-seq and the predicted target genes of miR-184-3p. **b** Heatmap of 12 mRNAs screened from (**a**). **c** The expression of *Egr1* gene in M0-T-exo and M0 macrophages detected by qRT-PCR. **d** The expression of EGR1 protein in M0-T-exo and M0 macrophages determined by IF staining. Cell nuclei were blue. Rac1 was red. **e** MFI of Rac1 in (**d**) calculated by ImageJ software. **f** The expression of *Egr1* gene in macrophages transfected with N.C. or miR-184-3p mimics detected by qRT-PCR. **g** The expression of EGR1 protein in macrophages transfected with N.C. or miR-184-3p mimics determined by IF staining. Cell nuclei were blue. Rac1 was red. **h** MFI of Rac1 in (**g**) calculated by ImageJ software. Western blot assays for p-JNK expression in M0-T-exo **(i)** and macrophages transfected with miR-184-3p mimics **(j)**. Data were expressed as mean ± SD (n = 3, ***p < 0.001)

To deeply explore the mechanism of exosomal miR-184-3p in the induction of macrophage polarization, mRNA sequencing (mRNA-seq) analysis was performed to determine the differentially expressed mRNAs in M0 macrophages and M0-T-exo, and the potential miR-184-3p targets were predicted by the online bioinformatic tool ENCORI. M0 macrophages were used as control to screen down-regulated genes in M0-T-exo, and combined with the predicted target gene profile of miR-184-3p, a total of 21 overlapping genes were screened out (Fig. 3a). With the fragments per kilobase of exon model per million mapped reads (FPKM) value greater than 10 as the standard, 12 possible regulatory molecules were further screened out (Fig. 3b). Combining the expression level and the difference in expression between the two groups, we focused on *Egr1* gene. More importantly, EGR1 protein has been reported to activate the JNK signaling pathway, a key signaling pathway that regulates macrophage polarization [26, 27]. Therefore, we concluded that overexpression of miR-184-3p in M0-T-exo inhibited JNK signaling pathway by down-regulating the expression of EGR1, thus promoting M2 polarization of macrophages.

To confirm the predicted regulatory mechanism, the expression of related genes and proteins were determined. Compared with M0 macrophages, the expression of *Egr1* gene and EGR1 protein in M0-T-exo detected by qRT-PCR (Fig. 3c) and IF staining (Fig. 3d, e) was significantly down-regulated. The same trend was also observed in macrophages transfected with miR-184-3p mimics (Fig. 3f-h). Accordingly, the expression of phospho-JNK (p-JNK) in M0-T-exo (Fig. 3i) and macrophages transfected with miR-184-3p mimics (Fig. 3j) measured by western blot was inhibited. The results of semi-quantitative analysis of western blot also confirmed this point (Additional file 1: Fig. S5). In sum, miR-184-3p in macrophages transferred by T-exo could inhibit the JNK signaling pathway by targeting EGR1, thereby inducing M2 polarization of macrophages.

### MiR-184-3p functioned as a Tumor suppressor by down-regulating MAML1

**Fig. 4:**
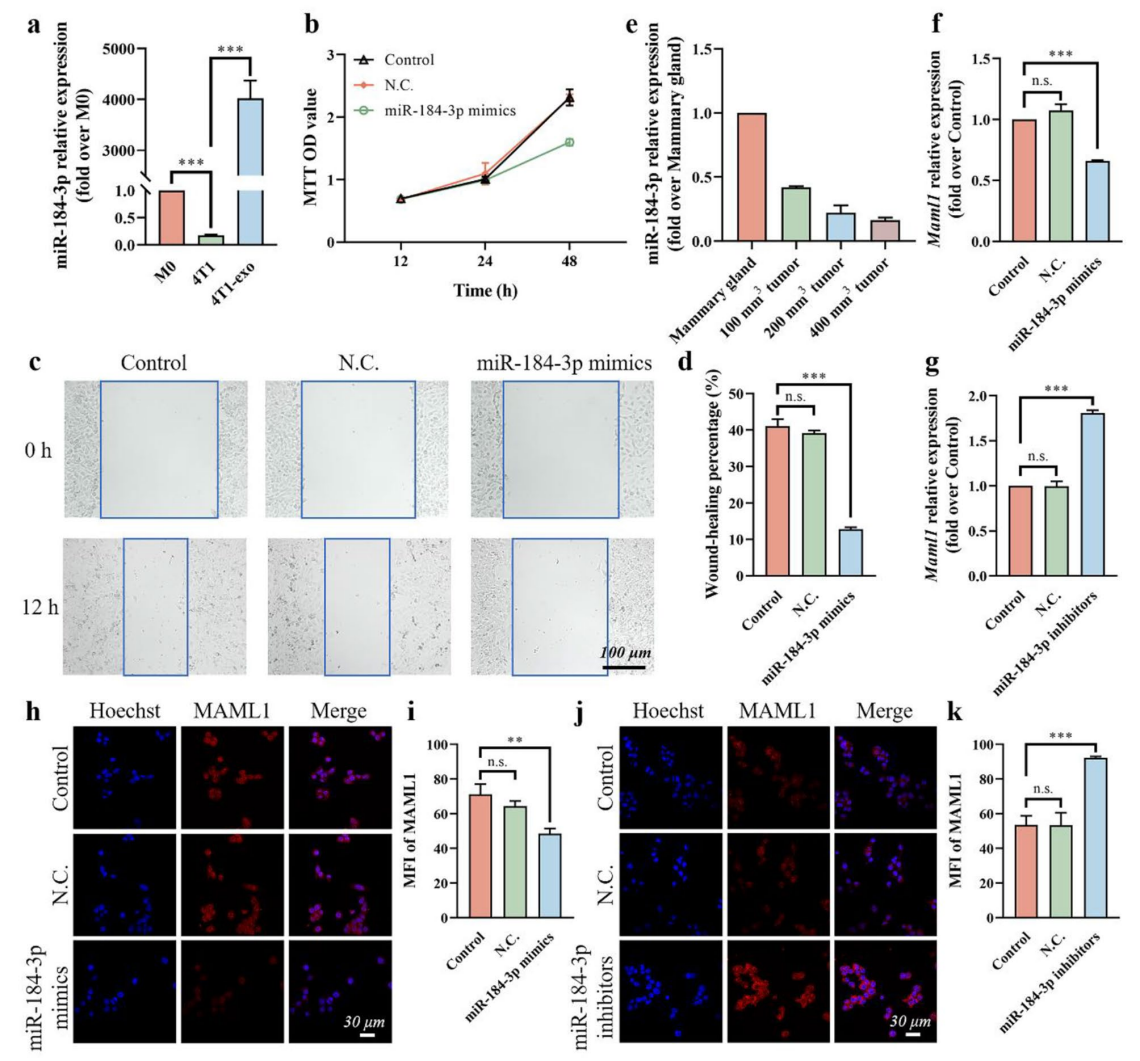
MiR-184-3p functioned as a tumor suppressor by down-regulating MAML1. **a** The expression of miR-184-3p in M0 macrophages, 4T1 cells and T-exo detected by qRT-PCR. **b** The proliferation of 4T1 cells transfected with N.C. or miR-184-3p mimics determined by MTT assay at different time points. Representative images of the wound-healing assay **(c)** and wound-healing percentage **(d)** of 4T1 cells transfected with N.C. or miR-184-3p mimics. **e** The expression of miR-184-3p in mammary glands and tumors of different sizes measured by qRT-PCR. The gene expression of *Maml1* in 4T1 cells transfected with miR-184-3p mimics **(f)** or miR-184-3p inhibitors **(g)** determined by qRT-PCR. The protein levels of MAML1 in 4T1 cells transfected with miR-184-3p mimics **(h)** or miR-184-3p inhibitors **(j)** determined by IF staining. Cell nuclei were blue. MAML1 was red. **i** MFI of MAML1 in (**h**) calculated by ImageJ software. **k** MFI of MAML1 in (**j**) calculated by ImageJ software. Data were expressed as mean ± SD (n = 3, **p < 0.01, ***p < 0.001)

The miRNA profiles of parent cells and their derived exosomes are often different. The expression of miR-184-3p in 4T1 and T-exo was measured by qRT-PCR, and we were surprised to find that although miR-184-3p was highly expressed in T-exo, the expression in 4T1 cells was very low (Fig. 4a). Combined with the previous findings, we speculated that miR-184-3p served as a tumor suppressor in 4T1 cells, which could be actively secreted out through exosomes. To investigate the role of miR-184-3p in 4T1 cells, we up-regulated or down-regulated the expression of miR-184-3p by transfecting miR-184-3p mimics (Additional file 1: Fig. S6a) or miR-184-3p inhibitors (Additional file 1: Fig. S6b), respectively. The proliferation ability of 4T1 cells was measured by MTT assay at different time points, and the results (Fig. 4b) showed that the highly expressed miR-184-3p could effectively inhibit the proliferation of tumor cells. Meanwhile, the results of the wound-healing assay showed that the migration ability of 4T1 cells was also inhibited by the overexpression of miR-184-3p (Fig. 4c, d).

In vivo results further confirmed the previous findings, as the level of miR-184-3p was significantly lower in tumor tissues than in normal mammary glands and were negatively correlated with tumor size (Fig. 4e). What’s more, the expression of miR-184-3p of the tumor tissues excised from the mice implanted with 4T1 cells mixed with conditioned macrophages conducted above was evaluated by qRT-PCR (Additional file 1: Fig. S7a, b). The larger tumors generated by 4T1 cells mixed with T-exo-polarized macrophages or miR-184-3p mimics-transfected macrophages had extremely low expression of miR-184-3p. Pretreating macrophages with JQ1 or Ex 26-CSOPOSA/JQ1 could rescue the expression of miR-184-3p.

In order to explore the molecular mechanism, we focused on *Maml1*, a potential target of miR-184-3p with strict stringency predicted by ENCORI. More importantly, MAML1, as a major transcriptional co-activator for Notch signaling, activates multiple signaling pathways that are dysregulated in tumor cells, including p53, NF-κB, and Wnt/β-catenin, to promote tumor development [28–31]. Therefore, the gene expression of *Maml1* and the protein levels of MAML1 in 4T1 cells transfected with miR-184-3p mimics or miR-184-3p inhibitors were determined respectively by qRT-PCR (Fig. 4f, g) and IF staining (Fig. 4h-k). The results showed that the gene expression of *Maml1* and the protein levels of MAML1 in 4T1 cells were decreased after transfection with miR-184-3p mimics and increased after transfection with miR-184-3p inhibitors. All these results indicated that miR-184-3p functioned as a tumor suppressor in TNBCs by targeting MAML1.

### HnRNPA2B1 directly mediated mir-184-3p packaging into Tumor cell-derived exosomes

**Fig. 5:**
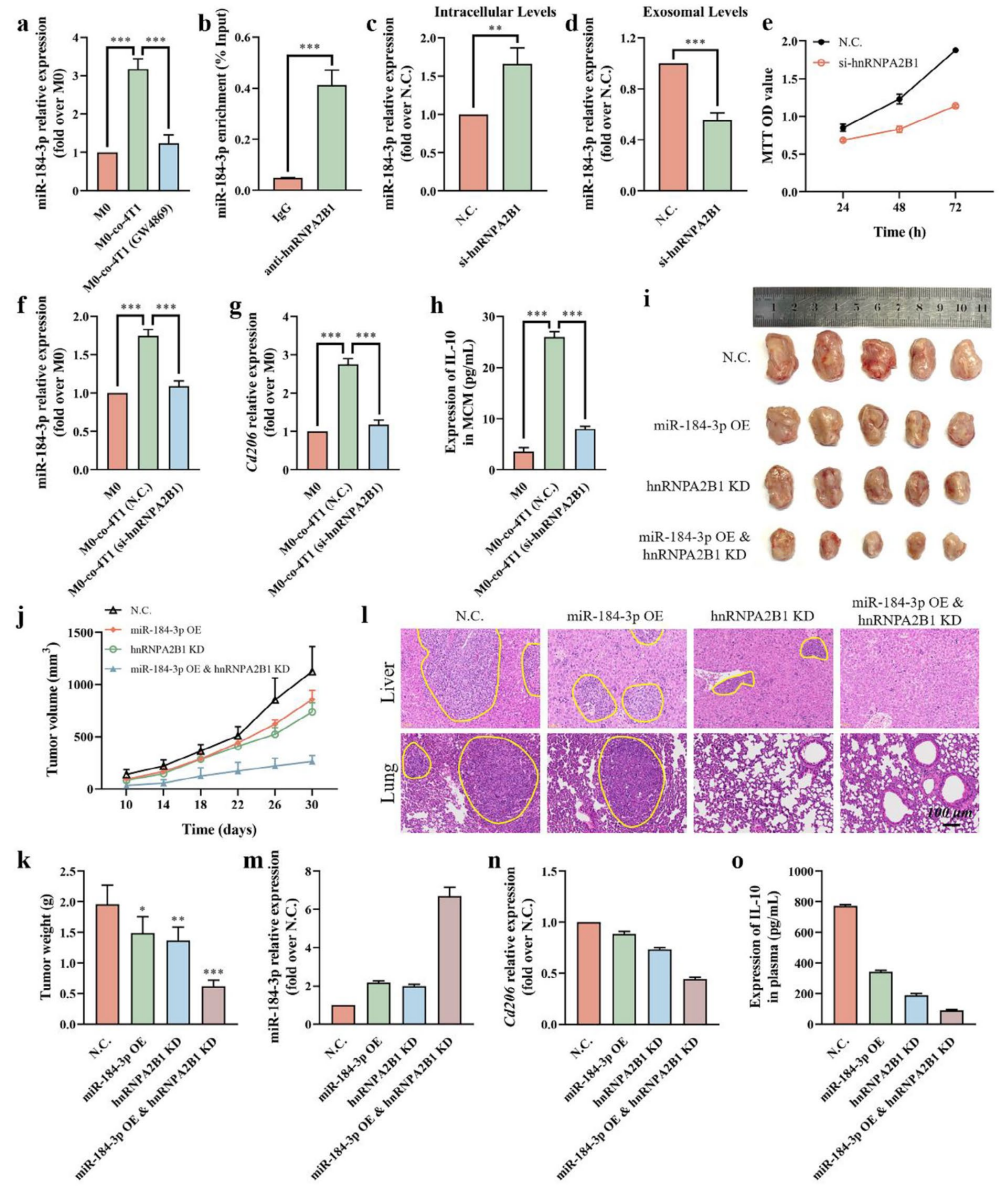
HnRNPA2B1 directly mediated miR-184-3p packaging into tumor cell-derived exosomes. **a** The expression of miR-184-3p in macrophages co-cultured with 4T1 cells pre-treated with or without GW4869 by qRT-PCR (n = 3). **b** RIP analysis of miR-184-3p bound by hnRNPA2B1 by incubating 4T1 cell lysates with IgG or anti-hnRNPA2B1 (n = 3). Intracellular **(c)** and exosomal **(d)** levels of miR-184-3p in 4T1 cells transfected with N.C. or si-hnRNPA2B1 (n = 3). **e** The proliferation of 4T1 cells transfected with N.C. or si-hnRNPA2B1 determined by MTT assay at different time points (n = 3). The expression of miR-184-3p **(f)** and *Cd206***(g)** in macrophages co-cultured with 4T1 cells transfected with N.C. or si-hnRNPA2B1 by qRT-PCR (n = 3). **h** ELISA for the secreted IL-10 in MCM (n = 3). Image of tumors **(i)**, tumor volume changes **(j)**, and tumor weight **(k)** of each group (n = 5). **l** H&E staining for livers and lungs from tumor-bearing mice. Tumor metastases were marked by yellow lines. The expression of miR-184-3p **(m)** and *Cd206***(n)** in tumor tissues detected by qRT-PCR (n = 3). **o** ELISA for the secreted IL-10 in plasma from tumor-bearing mice (n = 3). Data were expressed as mean ± SD (*p < 0.05, **p < 0.01, ***p < 0.001)

A co-culture assay was performed to determine whether miR-184-3p was mainly secreted by exosomes. The expression of miR-184-3p in macrophages co-cultured with 4T1 cells pre-treated with or without the exosome secretion inhibitor GW4869 was evaluated by qRT-PCR (Fig. 5a). Compared with M0 macrophages, the expression of miR-184-3p in M0 macrophages co-cultured with 4T1 cells (M0-co-4T1) was significantly increased. The pretreatment of GW4869 significantly lowered the delivery of miR-184-3p to M0 macrophages, and the expression of miR-184-3p in M0 macrophages co-cultured with 4T1 cells pre-treated with GW4869 (M0-co-4T1 (GW4869)) was similar to that in M0 macrophages. This result indicated that miR-184-3p was mainly delivered through exosomes.

Since miRNAs can be selectively sorted into exosomes through purposeful rather than passive processes, we explored the molecular mechanism that controlled the sorting of miR-184-3p into exosomes. RNA-binding proteins (RBPs) are a class of key molecules that contribute to the sorting process by recognizing specific motifs on miRNAs [32]. Based on the sequence of miR-184-3p, we focused on the RNA-binding protein hnRNPA2B1, as it has been shown to specifically bind to GGAG and AGG motifs [22, 33], both of which are included in the miR-184-3p sequence (UGGACGGAGAACUGAUAAGGGU). Specific binding of hnRNPA2B1 to miR-184-3p was verified by RNA immunoprecipitation (RIP) of hnRNPA2B1 from 4T1 cell lysates (Fig. 5b). The results showed that miR-184-3p detected by qRT-PCR was mainly amplified from hnRNPA2B1 polyclonal antibody (anti-hnRNPA2B1) immunoprecipitates rather than immunoglobulin G (IgG) immunoprecipitates, demonstrating specific binding of hnRNPA2B1 and miR-184-3p in cells.

We further evaluated the effect of hnRNPA2B1 knockdown (Additional file 1: Fig. S8) on miR-184-3p sorting into exosomes. After hnRNPA2B1 knockdown in 4T1 cells transfected with hnRNPA2B1 siRNA (si-hnRNPA2B1), miR-184-3p increased significantly in cells but decreased in exosomes (Fig. 5c, d). Consistent with the previously discovered function of miR-184-3p, the knockdown of hnRNPA2B1 effectively inhibited the proliferation of 4T1 cells by increasing intracellular miR-184-3p (Fig. 5e). Correspondingly, M0 macrophages co-cultured with 4T1 cells with low hnRNPA2B1 expression received less miR-184-3p from T-exo (Fig. 5f), thereby the M2 polarization process was blocked, as *Cd206* and secreted IL-10 were significantly reduced (Fig. 5g, h).

The role of miR-184-3p and hnRNPA2B1 in tumor growth and metastasis was further assessed in vivo. The volume of tumors generated by 4T1 cells transfected with different lentivirus and eventual tumor weight were monitored (Fig. 5i-k). The overexpression of miR-184-3p (miR-184-3p OE) or knockdown of hnRNPA2B1 (hnRNPA2B1 KD) could inhibit the growth of tumors to a certain extent, and the smallest and lightest tumors were present in the miR-184-3p OE & hnRNPA2B1 KD group. H&E staining was performed to observed the tumor metastases (marked by yellow lines) in the livers and lungs (Fig. 5l). Compared with the N.C. group, fewer metastases were observed in the miR-184-3p OE group and hnRNPA2B1 KD group, and more importantly, no metastasis occurred in the miR-184-3p OE & hnRNPA2B1 KD group. What’s more, increased expression of miR-184-3p (Fig. 5m) and decreased expression of *Cd206* (Fig. 5n) in tumor tissues in the miR-184-3p OE & hnRNPA2B1 KD group were confirmed by qRT-PCR, and the secretion of IL-10 detected by ELISA was also significantly reduced (Fig. 5o). All these results indicated that hnRNPA2B1 played an important role in the sorting of tumor suppressor miR-184-3p into exosomes, and blocking this process could effectively inhibit tumor growth and metastasis.

## Discussion

Due to the lack of specific treatment guidelines for this subtype, TNBCs are treated with standard therapy. However, this treatment has associated them with high rates of local and systemic recurrence [34]. There are few systemic treatment options other than chemotherapy, as the heterogeneity of TNBCs limits the successful development of targeted therapies [35]. Currently, there is no approved targeted therapy for TNBCs, and the molecular mechanisms driving the progression of TNBCs are still poorly understood.

Different components in TME, such as suppressive immune cells, altered extracellular matrix and certain soluble factors, synergistically hinder anti-tumor therapies and promote the progression and metastasis of breast cancer. TNBCs are considered to have a unique microenvironment different from other subtypes, which are characterized by the high expression of tumor-promoting factors and the infiltration of a large number of TAMs. TAMs originate from blood monocytes and are recruited to the tumor site by factors secreted by tumor or stromal cells. Breast cancer cells secrete factors that drive the polarization of macrophages to a tumor-promoting phenotype, and this effect is most pronounced in TNBCs [36, 37]. High levels of infiltrating TAMs are associated with a poor prognosis of TNBCs.

Exosomes play an important role in signal transduction. Therefore, we further explored the molecular mechanism of exosome-mediated macrophage polarization in TNBCs. Our results indicated that miR-184-3p is a key molecule in this process, and JQ1-loaded nanoparticles could reduce the delivery of miR-184-3p to macrophages by inhibiting the uptake of exosomes. At the same time, we found that the expression levels of miR-184-3p in cells and exosomes were significantly different, and miR-184-3p was identified as a tumor-suppressive miRNA with low expression in TNBCs. We fully explored the role of miR-184-3p in TNBCs. These results revealed that tumor cells selectively exported suppressive miRNAs *via* exosomes to promote tumor cell growth and metastasis, and remodel the immunosuppressive microenvironment. Although we focused here on the role of tumor suppressor miR-184-3p, the functions of other miRNAs should not be excluded. In fact, all encapsulated miRNAs might be involved in this process.

In previous studies, the role of a variety of miRNAs in tumor cells has been studied [38, 39]. The anti-tumor function was usually achieved by ectopic activation of suppressive miRNAs in tumor cells, but the active efflux of tumor cells through exosomes was ignored [40, 41]. Therefore, the mechanism of specific miRNA sorted into exosomes is a key point. So far, it has been described specific miRNAs are sorted into exosomes through different pathways and molecules, including binding to RBPs, ceramide signaling regulation, and mRNA-miRNA interactions, among which RBPs have been widely confirmed [42]. HnRNPA2B1 is one such RBP that can bind to miRNAs containing specific sequences to control their loading into exosomes. Here we found that hnRNPA2B1 at least selectively sorted miR-184-3p into exosomes through the GGAG and AGG motifs in the miRNA. Increasing the expression of miR-184-3p in tumor cells while down-regulating hnRNPA2B1 to block its secretion through exosomes could most effectively inhibit tumor growth and metastasis.

In this study, we demonstrated for the first time that tumor cells actively sorted the tumor-suppressive miR-184-3p into exosomes through hnRNPA2B1, and up-regulated the transcriptional co-activator MAML1 of multiple tumor-promoting signaling pathways, which was conducive to cell proliferation and metastasis. At the same time, miR-184-3p was delivered to macrophages through exosomes and induced the tumor-promoting phenotype of macrophages by inhibiting the JNK signaling pathway, thus synergistically promoting tumor progression. Oncogene *c-Myc* inhibitor inhibited T-exo uptake by macrophages by down-regulating Rac1 expression, thereby relieving the polarization function. These findings indicated that exosomes were important mediators for tumor cells to discard adverse factors and remodel a favorable niche, and revealed that miR-184-3p and hnRNPA2B1 represent the new therapeutic targets for TNBCs.

## Conclusions

In summary, our study has revealed that hnRNPA2B1 promoted tumor cell growth and metastasis *via* exosomal sorting of tumor suppressor miR-184-3p in TNBCs, while the secreted exosomes in turn function to remodel the immunosuppressive microenvironment by inducing M2 polarization of macrophages. Thus, miR-184-3p and hnRNPA2B1 represent the new therapeutic targets for TNBCs.

## Methods

### Cells and animals

Mouse breast cancer cell line 4T1 and mouse macrophage RAW264.7 (M0 macrophage) were purchased from the Cell Bank of Shanghai Institute of Biochemistry and Cell Biology, Chinese Academy of Sciences (Shanghai, China) and cultured in DMEM supplemented with 1% penicillin/streptomycin and 10% fetal bovine serum (FBS, v/v) in a humidified incubator with 5% CO_2_ at 37 ℃.

Female Balb/c mice (6 ~ 8 weeks) were purchased from the Shanghai Silaike Laboratory Animal Limited Liability Company. The experiments were performed in accordance with national regulations and was approved by Ethics Committee of the Institution on Laboratory Animal Care in Zhejiang University.

### Synthesis of Ex 26-CSOPOSA

The glycolipid grafts Ex 26-CSOPOSA were synthesized as previously described [23]. In brief, oxalyl chloride was reacted with stearyl alcohol (SA) in CH_2_Cl_2_ for 0.5 h, and then the reaction solution was reacted with p-hydroxybenzoic acid in the presence of triethylamine for 4 h. After removing excess alcohol and CH_2_Cl_2_ by distillation under reduced pressure, the intermediate product peroxalate-stearyl alcohol (PO-SA) with ROS-responsive peroxalate linkages was obtained after washing three times with diethyl ether. The ROS-sensitive glycolipid grafts (CSOPOSA) were obtained by a coupling reaction between carboxyl groups of PO-SA and amino groups of chitosan (CSO, 5.0 *k*Da) in the presence of 1-ethyl-3-(3-dimethylaminopropyl) carbodiimide (EDC) for 24 h.

To synthesize Ex 26 modified CSOPOSA, a certain amount of Ex 26, EDC and N-hydroxy succinimide were dissolved in DMSO and stirred for 2 h, then, NH_2_-PEG_2000_-NH_2_ and N, N’-disuccinimidyl carbonate were added successively and stirred for another 8 and 12 h. After that, the reaction solution was added dropwise into the CSOPOSA aqueous solution and stirred for another 24 h. Ultimately, Ex 26-CSOPOSA was obtained after dialysis and lyophilization.

### Isolation and characterization of exosomes

Exosomes were collected by density gradient ultracentrifugation according to the previously published protocol [43]. Briefly, the conditioned medium of 4T1 cells without FBS was centrifuged at 2,000×*g* for 20 min, followed by a centrifugation step of 10,000×*g* for 30 min. Subsequently, T-exo was obtained by ultracentrifugation twice at 100,000×*g* for 70 min. All centrifugations were carried out at 4 ℃. Measurement of the particle size was performed by a particle analyzer (Litesizer 500, Anton Paar, AT). TEM (Tecnai G2 spirit, ThermoFisher, USA) was used to observe the morphology of exosomes. Isolated exosomes resuspended in PBS were quantified using the BCA protein assay kit (P0010, Beyotime). Western blot was used to detect the levels of β-actin and exosome marker CD63 by using the corresponding cell lysate as a control (see below).

### Western blot analysis

Total protein was extracted from cells or exosomes using RIPA lysis buffer containing complete protease and phosphatase inhibitor cocktails and loaded onto SDS-PAGE gels. Then, protein in the gels was transferred onto polyvinylidene difluoride membranes. After being blocked with 5% bovine serum albumin (BSA), the membranes were incubated with primary antibodies overnight at 4 ℃, followed by incubation with the horseradish peroxidase-conjugated secondary antibody at room temperature for 2 h. Finally, the membranes were visualized with Omni-ECL™ Femto Light Chemiluminescence Kit (SQ201, Epizyme). The following primary antibodies were used: CD63 (ab217345, Abcam), β-actin (AF0003, Beyotime), Phospho-JNK (p-JNK, #4668T, Cell Signaling Technology), GAPDH (AF0006, Beyotime).

### Cell transfection

For transient transfection, cells seeded in culture plates were transfected with miR-184-3p mimics (sense-5’-UGGACGGAGAACUGAUAAGGGU-3’, antisense-5’-CCUUAUCAGUUCUCCGUCCAUU-3’), miR-184-3p inhibitors (sense-5’-ACCCUUAUCAGUUCUCCGUCCA-3’), hnRNPA2B1 siRNA (si-hnRNPA2B1, sense-5’-GGUGGCUUAAGCUUUGAAATT-3’, antisense-5’-UUUCAAAGCUUAAGCCACCTT-3’) or negative control (N.C.) by using transfection reagent lipofectamine 2000 (11,668,019, Invitrogen) according to the instructions. And the miR-184-3p overexpression lentivirus, hnRNPA2B1 knockdown lentivirus and N.C. lentivirus were synthesized by GenePharma (Shanghai, China) for stable transfection.

### Evaluation of M2 polarization of macrophages

RAW264.7 cells stimulated by T-exo or transfected with miR-184-3p mimics were collected and marked with Alexa Fluor 647-labeled anti-mouse CD206 monoclonal antibody after being blocked by 2% BSA in PBS. In addition, cells were pre-treated with JQ1 or Ex 26-CSOPOSA/JQ1 at a concentration of 0.5 µg/mL for 24 h before T-exo stimulation to investigate the drug intervention on the polarization process. The levels of CD206 on RAW264.7 cells were assayed by FCM using a Flow Cytometer (CytoFLEX LX, Beckman Coulter, USA). At the same time, the MCM was collected after the corresponding treatment and the secretion of IL-10 was detected by ELISA using the IL-10 ELISA Kit (EK210/4–96, MultiSciences) according to the manufacturer’s instructions.

### Cell proliferation and migration assays

Cell proliferation ability was assessed by MTT assay. In brief, after MCM-stimulated 4T1 cells or 4T1 cells transfected with miR-184-3p mimics or si-hnRNNPA2B1 in 96-well plates were exposed to MTT solution (5 mg/mL) for 4 h, DMSO was added to dissolve the formed formazan crystals, and the optical density (OD) at 570 nm was measured by a microplate reader (Model 680, Bio-Rad, USA).

The transwell assay was performed to investigate the effect of macrophages on tumor cell migration by using a 24-well plate with transwell inserts (8 μm pore size, Corning, USA). 4T1 cells (1 × 10^4^ cells/well) suspended in 100 µL serum-free DMEM were seeded in the upper chamber of transwell inserts, and M0 macrophages (2 × 10^5^ cells/well) pre-treated with T-exo or miR-184-3p mimics as described above were seeded in the lower chamber. After incubation for 24 h, 4T1 cells on the lower surface of inserts were stained with 0.1% crystal violet solution (w/w) and viewed by an optical microscope (Leica Microsystems, GER) to count the number of invasive cells. In addition, the migration ability of 4T1 cells transfected by miR-184-3p mimics was evaluated by wound-healing assay. When cells formed a confluent monolayer, a wound was created by manually scraping with a 200 µL pipette tip and the wound width was observed and measured at 0 and 12 h.

### Cellular internalization of exosomes

Exosomes were labelled with green fluorescence probe PKH67 (MIDI67-1KT, Sigma-Aldrich) according to the protocol. After RAW264.7 cells in 6-well culture plates (5 × 10^5^ cells/well) were treated with JQ1 or Ex 26-CSOPOSA/JQ1 at a concentration of 0.5 µg/mL for 24 h, PKH67-labelled exosomes were added and further incubated for 2 h. Cell suspensions of each group were prepared and the positive percentage was determined by FCM. In addition, the BCA protein assay kit and fluorescence spectrophotometer were used to measure the protein content and MFI of PKH67 in the cell lysate for further verification. To investigate the mechanism of cellular internalization of exosomes, RAW264.7 cells were pre-incubated with 30 µM Nystatin, 10 µM Chlorpromazine or 5 µM Cytochalasin D for 0.5 h before adding exosomes.

### Immunofluorescence staining

Cells seeded in 24-well plates with glass coverslips (4 × 10^4^ cells/well) were fixed with 4% formaldehyde for 20 min and permeabilized with 0.2% Triton X-100 for 15 min. After being blocked with 2% BSA for 30 min, the cells were incubated with the primary antibodies overnight at 4 ℃ followed by washing and stained by incubation with fluorescently-labelled secondary antibody incubation at 37 ℃ for 2 h. The following primary antibodies were used: Rac1 (BS71440, Bioworld), EGR1 (22008-1-AP, Proteintech), MAML1 (GTX104748-S, GeneTex), hnRNPA2B1 (14813-1-AP, Proteintech). Hoechst33342 was added to label the nuclei. The confocal laser scanning microscope (Ix81-FV1000, Olympus, JP) was used to observe the samples, and MFI was quantified by ImageJ software.

### RNA sequencing and bioinformatic analysis

Total RNA was extracted from M0 macrophages and M0-T-exo using RNAiso Plus (9109, Takara). RNA sequencing library preparation and high-throughput sequencing were performed by LC Bio (Hangzhou, China). The miRNA-seq was performed on an Illumina Hiseq2000/2500 according to the manufacturer’s instructions and the mRNA-seq was performed on an Illumina Novaseq™ 6000 following the protocol. Expression data were normalized to FPKM. Significantly differentially expressed genes were defined by |log2FC| >= 1 and adjusted p-value < 0.05. Bioinformatic analysis was performed using the OmicStudio tools at https://www.omicstudio.cn/tool [44]. The online prediction tool ENCORI (The Encyclopedia of RNA Interactomes, http://starbase.sysu.edu.cn) was used to predict the potential targets of miR-184-3p [45].

### Quantitative real-time PCR

Total RNA was extracted by RNAiso Plus and 1 µg of total RNA was reverse transcribed to synthesize cDNA using PrimeScript™ RT Reagent Kit (RR037A, Takara). For qRT-PCR, Hieff qPCR SYBR Green Master Mix (11203ES08, Yeasen) was used according to the manufacturer’s instructions. GAPDH was used to normalize the expression of mRNA. The expression of miR-184-3p was quantified using the Bulge-Loop miRNA qRT-PCR Primer Set (RiboBio, Guangzhou, China) and normalized to U6. The gene-specific primers sequence:

*Gapdh* (F—5’-CCTCGTCCCGTAGACAAAATG-3’, R—5’-TGAGGTCAATGAAGGGGTCGT-3’), *Cd206* (F—5’-AGGGAAACAATACCTTGAACCCAT-3’, R—5’-GAGATGGGAGAAGATGAAGTCAA-3’), *Egr1* (F—5’-AGCCGAGCGAACAACCCTATGAGC-3’, R—5’-CCAGGGAGAAGCGGCCAGTATAGGT-3’), *Maml1* (F—5’-GCCCGGTCGCGCATCTTCAT-3’, R—5’-GGGGCGAGGCAGGAGGAAGC-3’).

### Co-culture assay

4T1 cells pre-treated with GW4869 (10 µM) or pre-transfected with si-hnRNPA2B1 were seeded in the upper chamber of transwell inserts (0.4 μm pore size), while M0 macrophages were placed in the lower chamber. After co-incubation for 24 h, macrophages and MCM were collected. The expression of miR-184-3p and *Cd206* was determined by qRT-PCR, and the secretion of IL-10 was detected by ELISA.

### RNA immunoprecipitation

RNA immunoprecipitation (RIP) was performed using the RIP Kit (Bes5101, BersinBio) according to the manufacturer’s instructions. In brief, 4T1 cells were collected and lysed with polysome lysis buffer supplemented with protease inhibitor and RNase inhibitor for 20 min. After removing the DNA, cell lysate was incubated with 5 µg anti-hnRNPA2B1 or an equivalent amount of IgG at 4 ℃ for 16 h. Part of the untreated cell lysate (Input) was stored for later use. Then, the well-balanced protein A/G beads were added and incubated for another 1 h. After repeated washing with polysome washing buffer, co-immunoprecipitated RNA was extracted and analyzed by qRT-PCR.

### Evaluation of xenograft tumors in mice

4T1 cells mixed with the conditioned macrophages stimulated by T-exo or transfected by miR-184-3p mimics in a ratio of 1:3 were inoculated in situ in Balb/c mice (5 mice/group) to monitor tumor growth. After 25 d, all mice were sacrificed, and the tumor tissues were excised to measure the expression of miR-184-3p and *Cd206*.

To identify the role of miR-184-3p and hnRNPA2B1 in tumor growth and metastasis, 4T1 cells were stably transfected with N.C., miR-184-3p overexpression lentivirus (miR-184-3p OE), hnRNPA2B1 knockdown lentivirus (hnRNPA2B1 KD) or simultaneously transfected with miR-184-3p overexpression lentivirus and hnRNPA2B1 knockdown lentivirus (miR-184-3p OE & hnRNPA2B1 KD). Then, the transfected 4T1 cells were inoculated in situ in Balb/c mice (5 mice/group) to monitor tumor growth. After 30 d, all mice were sacrificed to measure the expression of miR-184-3p and *Cd206* in excised tumors and the secretion of IL-10 in plasma. The livers and lungs were fixed in 4% paraformaldehyde and embedded into paraffin for H&E staining.

### Statistical analysis

All results were expressed as mean ± standard deviations (SD). Statistical significance was calculated by two-tailed Student’s t-test. The statistical significance was defined as a P-value less than 0.05.

### Electronic supplementary material

Below is the link to the electronic supplementary material.

Supplementary Material 1

## Data Availability

The data used or analyzed during this study are available from the corresponding author on reasonable request.
