# Supplementary material for: Intervening in hnRNPA2B1-mediated exosomal transfer of tumor-suppressive miR-184-3p for tumor microenvironment regulation and cancer therapy
Source: J Nanobiotechnology. 2023 Nov 14;21:422. doi: 10.1186/s12951-023-02190-w (PMC10644646; doi:10.1186/s12951-023-02190-w)
Supplement: Supplementary file 1 — Supplementary Material 1 [file 12951_2023_2190_MOESM1_ESM.docx]

**Additional file 1**

**Intervening in hnRNPA2B1-mediated exosomal transfer of tumor-suppressive miR-184-3p for tumor microenvironment regulation and cancer therapy**

Xueqing Zhou ^1^, Yiling Hong ^1^, Yupeng Liu ^1, 2^, Li Wang ^1^, Xuan Liu ^1^, Yi Li ^1^, Hong Yuan ^1, 3^, Fuqiang Hu ^1, 3 *^

^1^ *College of pharmaceutical science, Zhejiang University, Hangzhou 310058, China*

^2^ *Department of Clinical Pharmacology, Affiliated Hangzhou First People’s Hospital, School of Medicine, Zhejiang University, Hangzhou 310006, China*

^3^ *Jinhua Institute of Zhejiang University, Jinhua 321299, China*

^*^ Corresponding author: Fuqiang Hu. E-mail: [hufq@zju.edu.cn](mailto:hufq@zju.edu.cn); Tel/Fax: +86-571-88208439

**This PDF file includes：**

Fig. S1 to S8


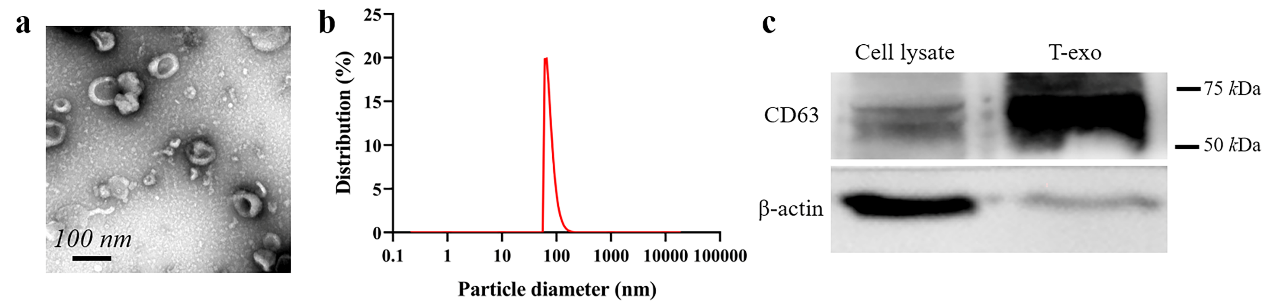


**Fig. S1** The TEM image **(a)**, size distribution **(b)**, and CD63 levels **(c)** of exosomes.


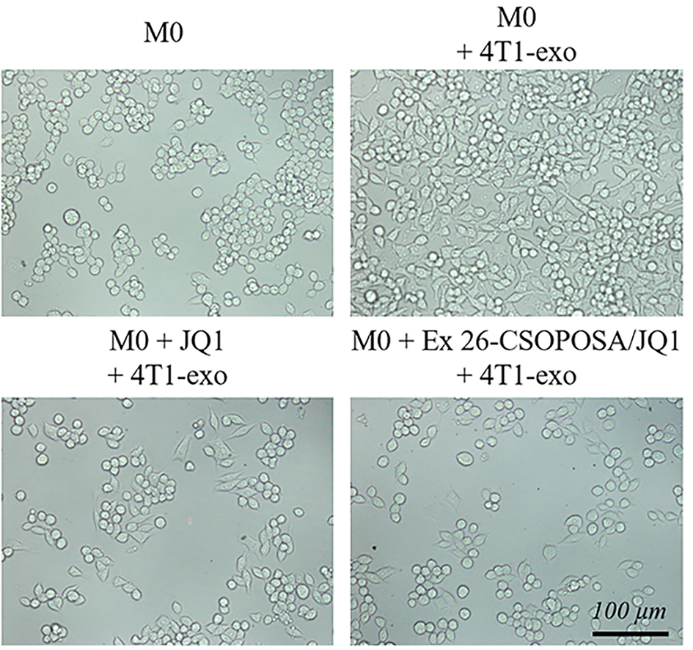


**Fig. S2.** Morphological changes of T-exo-stimulated macrophages with or without the pretreatment of JQ1 or Ex 26-CSOPOSA/JQ1.
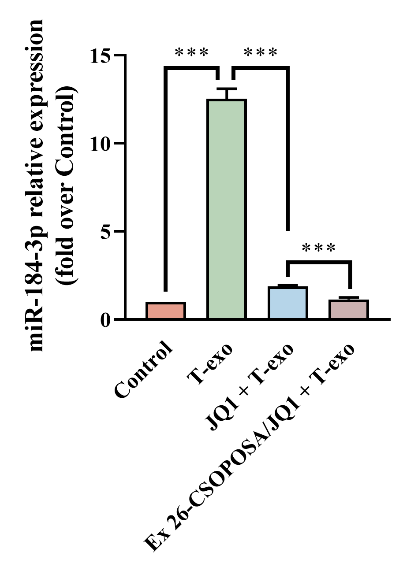


**Fig.** **S3** The expression of miR-184-3p in T-exo-stimulated macrophages with or without the pretreatment of JQ1 and Ex 26-CSOPOSA/JQ1 measured by qRT-PCR. Data were expressed as mean ± standard deviation (n = 3, ***p < 0.001).


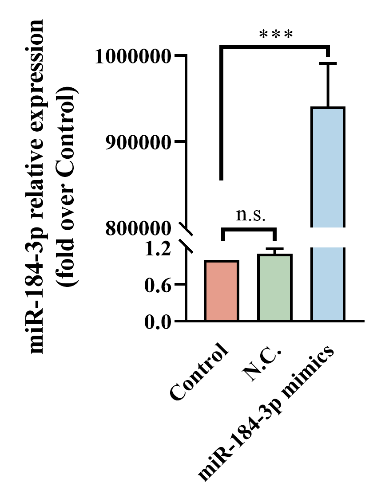


**Fig. S4** The expression of miR-184-3p in macrophages transfected with N.C. or miR-184-3p mimics measured by qRT-PCR. Data were expressed as mean ± standard deviation (n = 3, ***p < 0.001).


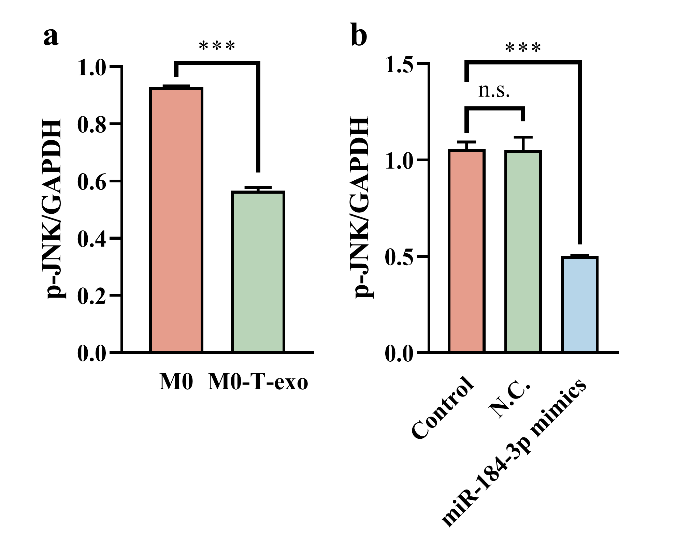


**Fig. S5 a** The semi-quantitative analysis of western blot in Fig. 3i by ImageJ software. **b** The semi-quantitative analysis of western blot in Fig. 3j by ImageJ software. Data were expressed as mean ± standard deviation (n = 3, ***p < 0.001).


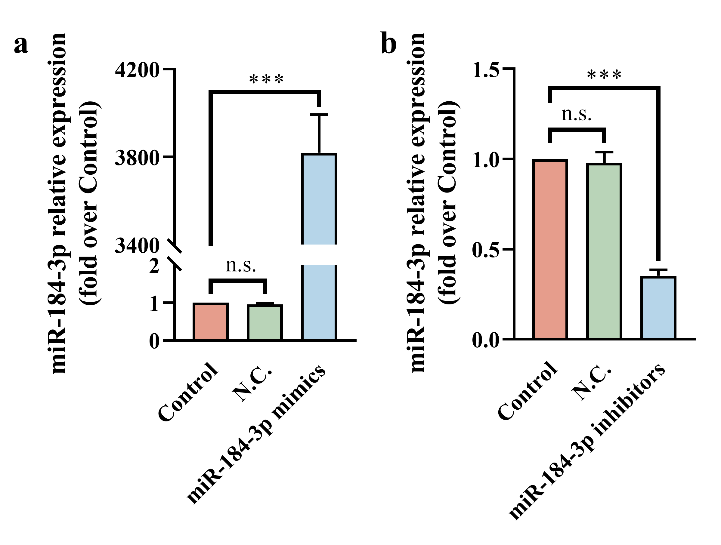


**Fig. S6** The expression of miR-184-3p in 4T1 cells transfected with miR-184-3p mimics **(a)** or miR-184-3p inhibitors **(b)** measured by qRT-PCR. Data were expressed as mean ± standard deviation (n = 3, ***p < 0.001).


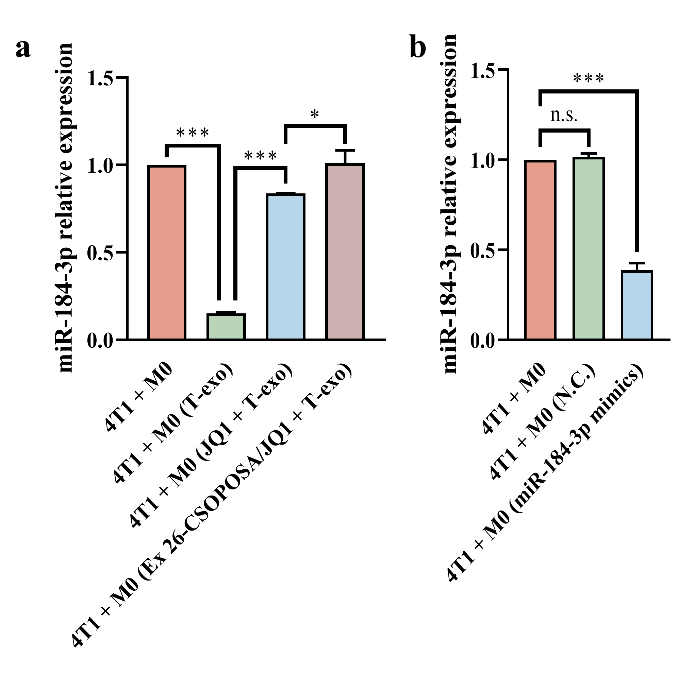


**Fig. S7** The expression of miR-184-3p in tumor tissues generated by 4T1 cells mixed with T-exo-stimulated macrophages **(a)** or miR-184-3p mimics-transfected macrophages **(b)** detected by qRT-PCR. Data were expressed as mean ± standard deviation (n = 3, *p < 0.05, ***p < 0.001).


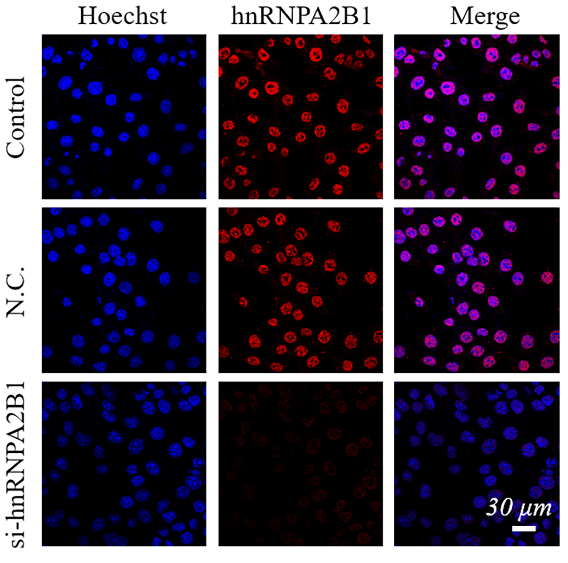


**Fig. S8** The expression of hnRNPA2B1 in 4T1 cells transfected with si-hnRNPA2B1 or N.C. determined by IF staining. Cell nuclei were blue. HnRNPA2B1 was red.
